# A Wind Tunnel Experimental Study of Icing on NACA0012 Aircraft Airfoil with Silicon Compounds Modified Polyurethane Coatings

**DOI:** 10.3390/ma14195687

**Published:** 2021-09-29

**Authors:** Bartlomiej Przybyszewski, Rafal Kozera, Zuzanna D. Krawczyk, Anna Boczkowska, Ali Dolatabadi, Adham Amer, Bogna Sztorch, Robert E. Przekop

**Affiliations:** 1 Faculty of Materials Science and Engineering, Warsaw University of Technology, 141 Woloska Str., 02-507 Warsaw, Poland; rafal.kozera@pw.edu.pl (R.K.); zuzanna.krawczyk2.stud@pw.edu.pl (Z.D.K.); anna.boczkowska@pw.edu.pl (A.B.); 2 Technology Partners Foundation, 5A Pawinskiego Str., 02-106 Warsaw, Poland; 3 Department of Mechanical, Industrial and Aerospace Engineering, Concordia University, 1455 de Maisonneuve Blvd. W, Montreal, QC H3G 1M8, Canada; ali.dolatabadi@concordia.ca (A.D.); ad_ha_am@hotmail.com (A.A.); 4 Center for Advanced Technologies, Adam Mickiewicz University in Poznan, Uniwersytetu Poznanskiego 10, 61-614 Poznan, Poland; bognasztorch@gmail.com (B.S.); rprzekop@amu.edu.pl (R.P.)

**Keywords:** polyurethane nanocomposite coatings, anti-icing coatings, icing wind tunnel, silsesqioxane, spherosilicate

## Abstract

Ice formation on the aerodynamic surfaces of an aircraft is regarded as a major problem in the aerospace industry. Ice accumulation may damage parts, sensors and controllers and alter the aerodynamics of the airplane, leading to a range of undesired consequences, including flight delays, emergency landings, damaged parts and increased energy consumption. There are various approaches to reducing ice accretion, one of them being the application of icephobic coatings. In this work, commercially available polyurethane-based coatings were modified and deposited on NACA 0012 aircraft airfoils. A hybrid modification of polyurethane (PUR) topcoats was adopted by the addition of nanosilica and three-functional spherosilicates (a variety of silsesqioxane compound), which owe their unique properties to the presence of three different groups. The ice accretion on the manufactured nanocomposites was determined in an icing wind tunnel. The tests were performed under three different icing conditions: glaze ice, rime ice and mixed ice. Furthermore, the surface topography and wetting behavior (static contact angle and contact angle hysteresis) were investigated. It was found that the anti-icing properties of polyurethane nanocomposite coatings strongly depend on the icing conditions under which they are tested. Moreover, the addition of nanosilica and spherosilicates enabled the reduction of accreted ice by 65% in comparison to the neat topcoat.

## 1. Introduction

In various industries, such as aerospace, telecommunication, automotive or wind power plants, implemented materials are subjected to not only mechanical loads but also adverse weather conditions. One of the most important problems in aviation is ice formation on the aerodynamic surfaces, which may cause major issues, such as the alternation of the aerodynamics of an aircraft, jamming, damage of parts and sensors and even, in the case of thick ice layers, premature stall [1,2,3]. These may further lead to emergency landings, flight delays, the obligatory replacement of parts of the machine, increased consumption of energy and, as a result, restricted flight operations [4]. During the flight, supercooled water droplets are the main source of the accreted ice. When water droplets with a temperature below the freezing point strike the leading surfaces of the aircraft, they may freeze either immediately or after moving across the surface. Spreading may be caused by the relative velocity between the airplane and the surroundings [5]. The build-up of thick and heavy ice layers may be a result of the gradual accumulation of ice over time due to the consecutive impingement of multiple water droplets [6].

There are various methods of dealing with the icing problem, including mechanical or chemical ice removal as well as thermal and electrical deicing systems [7,8,9,10]. The other, presumably more optimal, solution is the application of anti-icing coatings on the surfaces susceptible to ice accretion [11,12,13].

Hydrophobic and superhydrophobic coatings are often investigated in terms of icephobicity [14]. Many polyurethane-based nanocomposite coatings, which were widely described in the literature [15,16,17], showed potential in anti-icing application as well. It was found that the hierarchical structure obtained by the addition of nanoparticles (e.g., SiO_2_, TiO_2_, ZrO_2_) together with a thin layer of highly hydrophobic components (e.g., fluorine compounds, stearic acid, polydimethylsiloxane (PDMS), etc.) rendered the surface superhydrophobic [18,19,20,21,22].

In the last 10 years, organosilicon compounds have drawn great attention due to their effectiveness in creating chemical structures with hydro- and icephobic properties [23]. Their special usefulness should be explained by the chemical structure and properties of functional groups, characterized by the presence of various organic groups and, on the other hand, silanol groups capable of forming permanent covalent bonds with the surface of steel, concrete, aluminum or alloys. It should be noted here that the role of these connections, resulting from the properties of functional groups and the structure of molecules, is important to understand from the point of view of designing new materials [24].

Recently, an increase in the surface tension modifying applications using organofunctional silsesquioxanes and their derivatives, e.g., spherosilicates, was observed. Moreover, their effectiveness in the formation of icephobic coatings was noted [25]. Polyhedral oligomeric silsesquioxanes make a group of organosilicon compounds of well-defined structure, most of them being described by a general formula [RSiO_1,5_]n, where R may be a hydrogen atom, an alkyl group, an aryl group and their derivatives. The character of this group is interesting due to the possibility of obtaining new, unique properties through various modification procedures and reactive or non-reactive substituents at the silicon atom located in the corner of the cage. Silsesquioxanes are hybrid compounds that combine the features of inorganic (siloxane core of silsesquioxanes) and organic (organic functional groups attached to the core structure) compounds. These reagents owe their popularity to a growing number of applications in various industries, mainly due to good thermal and mechanical properties, dispersion properties as well as the ease of their further functionalization towards desirable rheological or solubility properties [24,26]. 

Functionalized organosilicon compounds have hydrophobic, often superhydrophobic, properties and are successfully used as surface modifiers to obtain the desired characteristics [27]. Hydrophobic properties, as well as icephobic ones, depend to a large extent on the surface structure and its free energy. The ability to effectively repel water particles, prevent water condensation inside the structure and reduce the effect of ice adhering to the surface is related to the low free energy of the surface [28]. Silsesquioxanes derivatives are one of the most effective surface energy modifiers that, due to their multifunctional structure, can also effectively and permanently combine with the structure of the polymer matrix used [29].

Unfortunately, most of the cited works deal only with wetting properties. There are insufficient studies concerning PUR-based nanocomposite coatings with improved anti-icing properties. F. Carreno et al. [30] presented the two-step procedure for obtaining the icephobic coatings based on an organic-inorganic sol-gel applied over a modified commercial polyurethane-based paint that is fluoride and nanoparticle-free. The proposed modifications led to the reduction of the adhesion force of the ice up to 80% with respect to the original paint. In another work [31], the authors measured the water contact angle and sliding angle of in-house synthesized fluorinated polyurethane after exposure in −10 °C. It was presented that the superhydrophobicity of the coating was still achieved after 100 h at −10 °C. Nonetheless, the proposed technologies have several limitations, such as high costs or complicated preparation procedures. 

Moreover, the type of accumulated ice is an important factor in the measurement of anti-icing properties and thus the obtained results [32,33]. When water freezes in various atmospheric conditions, different types of ice are created. These different types of ice vary in microstructures and densities and behave in different manners when adhering to the investigated surface. The first one, glaze ice, is formed in relatively high temperatures below 0 °C, and it is usually dense, clear and smooth. The second one, rime ice, is formed in lower temperatures and is characterized by a white and feathery appearance; it is also less dense. Discrepancies in the properties of the icing types are caused mainly by differences in their forming. Water droplets turn into glaze ice immediately after contact with a surface. On the contrary, rime ice forms when some of the water droplets move along the surface before freezing, with some of them falling off before changing their state of matter. It is suggested that, due to its physical properties, glaze ice prohibits higher adhesion pressure and, hence, is harder to remove than rime ice. There is also mixed ice, which consists of both rime and glaze ice [34,35]. Various researchers have undertaken the challenge of coming up with prediction models of ice formation in different temperatures [36,37,38,39,40]; however, they are still far from perfect.

Among the applications of anti-icing surfaces, there is a need to remove several different types of ice depending on the icing conditions. As a result, it is a limitation of most research that only one type of ice is tested for anti-icing surfaces in each laboratory with the same measurement techniques [41,42]. 

In order to fill this gap in the literature, in this work, icing wind tunnel tests were carried out in three different temperatures where three different types of ice were accreted (glaze, rime and mixed). Moreover, to avoid edge effect and simulate realistic conditions, the NACA0012 aircraft airfoils were used in this study. Polyurethane coatings with anti-icing properties were modified by the addition of nanosilica and in-laboratory synthesized siloxane-based compounds and applied on NACA profiles by air gun spraying. Measurements of the wettability parameters (static contact angle and contact angle hysteresis) on the investigated surfaces were carried out to determine the relationship between hydrophobic and anti-icing properties. The surface topography was investigated to identify the relationship between roughness and the results obtained in the icing wind tunnel. It was found that the anti-icing properties of polyurethane nanocomposite coatings strongly depend on the icing conditions under which they are tested. Moreover, it was demonstrated that the relationship between hydrophobic and anti-icing properties is not as simple as presented in the literature so far (the “higher hydrophobicity, higher icephobicity” theory). As a result of the performed hybrid modifications of polyurethane chemical composition, the mass of accreted ice on the investigated NACA0012 profiles during the icing wind tunnel tests was significantly reduced (in some cases by around 65%). 

## 2. Materials and Methods

### 2.1. Materials

A three-component system PUR topcoat (Aviox^®^ finish 77702) was obtained from Akzo Nobel, Netherlands. It was used in combination with the Aerodur^®^ HS 37092 (Akzo Nobel, Sassenheim, The Netherlands) component amine cured epoxy primer. To modify the topcoat, in-laboratory synthesized POSS-based compounds were used (see chapter 3.1). Moreover, DC88, a silane/siloxane commercial blend (Dow Corning^®^, Midland, MI, USA), was utilized. The hydrophobic nano-silica Aerosil^®^ R805 (Evonik, Hanau, Germany) used in this study has a specific surface area of 125–175 m^2^/g and a nominal particle size of 12 nm. It was produced by treating fumed nano-silica with organosilane. All the commercially available materials were used as received. The modified PUR coatings were deposited on NACA0012 profiles made of 2024 aluminum alloy (WMH Group, Essen, Germany).

#### 2.1.1. Synthesis of Trifunctional POSS-Based Compounds 

The chemicals were purchased from the following sources: Octahydrospherosilicate was prepared according to the literature procedure [43]; olefins (vinyltrimethoxysilane, hexene) from Linegal Chemicals; allyl-2,2,3,3,4,4,5,5-octafluorophentyl ether was prepared according to the literature procedure [44]; solvents toluene from Avantor Performance Materials, Poland S.A. chloroform-d, toluene- d8, Karstedt catalyst from Sigma Aldrich, Toluene was dried and purified with MB SPS 800 Solvent Drying System and stored under argon atmosphere in Rotaflo Schlenk flasks.

*Synthesis of 1,3,5,7,9,11,13,15-tris (dimethyl (3-(2,2,3,3,4,4,5,5-octafluoropenthyloxy)propyl) siloxy)-tris ((hexyl)dimethylsiloxy)-bis ((trimethoxysilyl)ethyldimethylsiloxy)-pentacyclo[9.5.1.13,9.1 5,15.1 7,13]octasiloxane (POSS14).*

Vinyltrimethoxysilane (0.049 mol), hexene (0.074 mol) and allyl-OFP (0.074 mol) in molar ratio 2:3:3 were added to the solution of octaspherosilicate (25 g, 0.196 mol) in toluene. The mixture was constantly stirred and heated to 60–70 °C, then added to the system in an amount that varied from 8 × 10^−5^ eq. of Karstedt catalyst. Reaction mixture was heated in reflux and stirred until the full conversion of Si–H was detected by FT-IR and ^1^H NMR.

### 2.2. Preparation and Deposition of Silica/POSS14/Polyurethane (PUR) Nanocomposite Coatings

The 2024 aluminum alloy NACA0012 profiles were first cleaned in acetone. Initially, both primer components were mixed according to the manufacturer’s indications, then sprayed on Al profiles by means of a Walther^®^ Pilot XIII air spray gun (Walther, Wuppertal-Vohwinkel, Germany) and left to dry for 3 h. Meanwhile, PUR topcoats with 3 wt % content of nano-silica, POSS14 (5 wt %) and DC88 (5 wt %) were prepared by mixing the liquid additive with PUR using a magnetic stirrer. Before spraying, nano-silica suspensions on the modified PUR coating were prepared by means of an ultrasonic gun (VCX 750, Sonics and Materials Inc., Newtown, CT, USA) for 30 min. The suspensions were then sprayed on top of the already applied primer and left to dry for 24 h. The different compositions prepared are shown in Table 1.

### 2.3. Characterization

For the characterization of in-laboratory synthesized POSS-based compounds ^1^H and ^13^C{^1^H}, NMR spectra were recorded on a Bruker Ultrashield 300 MHz operating at 300 and 75 MHz, respectively. ^29^Si{^1^H} NMR spectra were recorded on a Bruker Ascend 400 MHz Nanobay operating at 79 MHz. Fourier transform infrared (FT-IR) spectra were recorded on a Nicolet iS50 Fourier transform spectrophotometer (Thermo Fisher Scientific, Waltham, MA, USA) equipped with a diamond ATR unit with a resolution of 0.09 cm^−1^. The spectra were collected in the 400–4000 cm^−1^ range; 16 scans were collected for each spectrum.

The coating thickness was measured by an Extech^®^ CG204 (Extech, Nashua, NH, USA) coating thickness tester at least six times across the sample using the eddy current measurement mode.

Wettability properties (static contact angle (SCA), advancing contact angle (ACA), receding contact angle (RCA), sliding angle (SA)) were measured by a contact angle measurement system (Data Physics, GmbH OCA 15, Filderstadt, Germany). All angles of each sample were measured at least three times across the sample surface using the sessile drop method by dispensing 3 µL (SCA), 5 µL (ACA, RCA) and 10 µL (SA) of deionized water on the sample’s surface. The contact angle hysteresis (CAH) was calculated as the difference between ACA and RCA.

Surface roughness was measured by means of a laser scanning confocal microscope (MarSurf CM Explorer, provided by Mahr, Göttingen, Germany). The test standard was ISO-25178. All the measurements were acquired on a 1550 µm × 1550 µm area with 0.67 µm spacing. Three different randomly selected areas were measured on each sample. The obtained parameters were S_a_ and S_z_, both of which are defined in detail by Zhang et al. [45].

**Figure 1:**
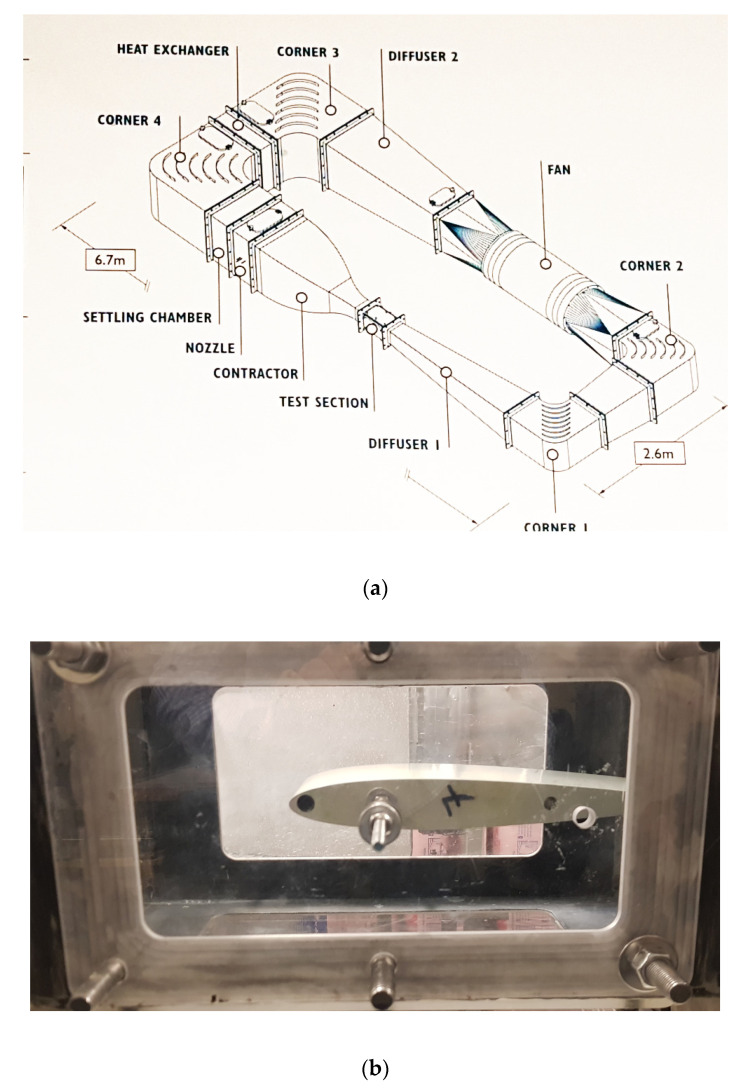
(**a**) Schematic of the icing wind tunnel; (**b**) NACA 0012 profile mounted in icing wind tunnel test section.

Ice accretion tests were performed by the use of icing wind tunnel (Figure 1). The icing wind tunnel was used to simulate real in-flight icing conditions by mimicking a cloud in the test section at sub-zero temperatures. The main variables that were controlled to simulate different environmental conditions were air speed, temperature and liquid water content (LWC). LWC is defined as mass of water in a given cloud per unit of dry air. The icing wind tunnel was of a closed-circuit type with a constant cross-sectional area of 20 cm× 20 cm and 40 cm in length. Air was driven by a centrifugal fan that was controlled by a variable motor. Air speed in the test section was 45 m/s. Sub-zero temperatures were achieved in the test section by the refrigeration unit and regulated through the PID controller. In presented work, tests were performed under three different temperatures to achieve different icing conditions: −5 °C (glaze ice), −10 °C (mixed ice), −15 °C (rime ice). These temperatures are experimentally proven to facilitate the formation of the chosen icing type [46]. The air speed and temperature were monitored downstream of the test section using a pitot tube and thermo-couples, respectively, placed at various positions. Up-stream of the test section, an atomizer spray was placed that sprayed fine mist that generated a cloud in the test section. The icing cloud properties and LWC were controlled by the air and water pressure regulating the spray. The LWC in the icing wind tunnel was 0.5 g/m^3^.

Ice reduction was calculated from the obtained ice accretion values using formula:

$$\mathrm{Ice}\;\mathrm{reduction}=100\%\times \left(1-\frac{\mathrm{accreted}\;\mathrm{ice}\;\mathrm{for}\;\mathrm{Sample}\;X}{\mathrm{accreted}\;\mathrm{ice}\;\mathrm{for}\;\mathrm{Reference}\;\mathrm{Sample}}\right)$$

## 3. Results

### 3.1. Synthesis of Trifunctional POSS-Based Compounds

**Figure 2:**
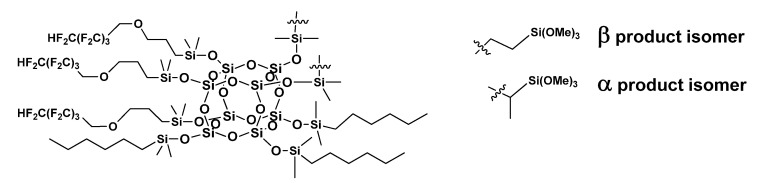
Synthesis of trifunctional POSS-based compound.

POSS-based compounds were prepared according to the synthesis procedure described in Section 2.1.1. They were investigated by NMR and FT-IR spectroscopy to prove the completion of the reactions (the disappearance of the characteristic signal was observed at 2141 and 889 cm^−1^, respectively, due to the stretching and bending of the Si-H group). For all of the compounds, the hydrosilylation proceeded with the completion of the reactions (~99%). The structure and purity of the modifiers were confirmed by NMR analysis. It was observed that the hydrosilylation of olefns proceeded selectively to the β-isomer, except for trimethoxysililethyl, where the formation of α-isormes was also observed in a proportion of ~21% in all of the cases. Due to the applied synthesis procedure, the distribution of the functional groups in the obtained compound was statistically complex. We obtained a mixture of compounds from the group distribution maximum for the structure in Figure 2.

The selectivity of the reaction, determined by NMR spectroscopy, showed that the product contained a mixture of trimethoxysililethyl **α** and **β** group in a 21:79 ratio.

**^1^H NMR** (400 MHz, CDCl_3_): δ (ppm) = 6.19 − 5.93 (m, 3H, -CF_2_H), 3.90 (t, J = 14.1 Hz, 6H, O-CH_2_-CF_2_), 3.58–3.53 (s, OMe, 18H), 1.68–1.61 (m, 6H, O-CH_2_-CH_2_-CH_2_-Si), 1.30–1.27 (m, 24H, hexyl -CH_2_- groups), 1.13 (d, J = 7.5 Hz, 3H, alpha product -CH_3_), 0.89–0.86 (t, J = 6.6 Hz, 9H, -CH-_2_CH_3_), 0.60 (m, 20H, -CH_2_-CH_2_-CH_2_-Si, SiCH_2_CH_2_Si), 0.15, 0.14, 0.12 (s, 48H, SiMe_2_);

**^13^C NMR** (101 MHz, CDCl_3_): δ (ppm) = 107.78 (tt, J_1_ = 253.6 Hz, J_2_ = 30.6 Hz, -CF_2_-), 75.77 (O-CH_2_-CH_2_-CH_2_-Si), 67.64 (t, J = 25.8 Hz, O-CH_2_-CF_2_), 50.68 (OMe), 33.16, 31.73 (hexyl), 23.16 (O-CH_2_-CH_2_-CH_2_-Si), 23.02, 22.74, 17.77, 14.25 (hexyl), 13.52 (O-CH_2_-CH_2_-CH_2_-Si), 8.60 (Si-CH_2_CH_2_-Si), 7.37, 5.27 (SiCH (CH_3_)Si), 0.42 (Si-CH_2_CH_2_-Si), 0.22 (hexyl SiMe_2_), −0.38 (fluorinated group SiMe_2_), −1.05 (trimethoxysilylethyl group SiMe_2_);

**^29^Si NMR** (795 MHz, CDCl_3_): δ (ppm) = 13.37 − 12.73 (SiMe_2_), −41.61 (Si (OMe)_3_), −108.90–(−109.07) (core);

**^19^F NMR** (3765 MHz, CDCl3): δ (ppm) = −119.90, −125.90, −130.57, −137.46, −137.46 (d, J = 52.4 Hz).

### 3.2. Coating Thickness and Roughness

The coating thickness was measured as described in a previous paper [47]. The results were the same for each tested sample: the thickness of the primer layer was 25±3 μm and that of the whole system was 55±5 μm. The average roughness parameters for each sample are presented in Table 2.

It can be seen that chemical modification increases the roughness of the material. The difference is quite small for Sample 5, whereas it is the most apparent for Sample 2. Sample 4 exhibits low values of $S_{a}$; however, its value of $S_{z}$ is one of the highest obtained. The samples modified with two compounds are rougher than the samples with sole DC88 or POSS14 added.

**Figure 3:**
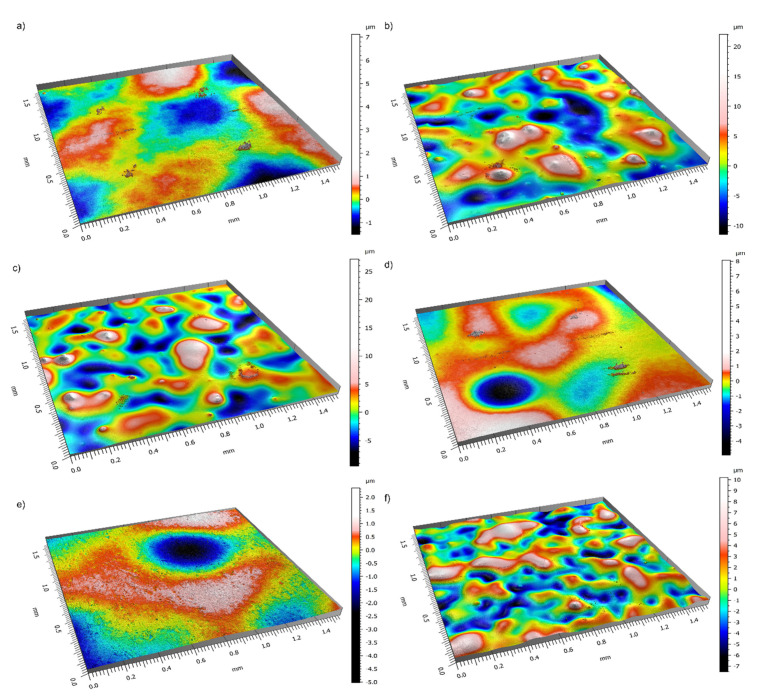
Profiles of the samples (**a**) Reference (**b**) SiO_2_ + DC88 (**c**) SiO_2_ (**d**) DC88 (**e**) POSS14 (**f**) SiO_2_ + POSS14.

Looking at the standard deviation of the measurements, it seems that the average height was similar in different areas of the samples; nevertheless, there are quite significant differences in the values of maximum height. To visualize the presented results, selected profiles for each sample are shown in Figure 3.

It should be noted that, in the profiles shown in Figure 3a,d,e, the areas of the same height are much broader than in the rest of the profiles. The highest and lowest areas have quite regular shapes. On the other hand, in the profiles shown in Figure 3b,c,f, such areas are quite small and irregular but more raised and, overall, these profiles are more convex. The observations are in agreement with the data presented in Table 2.

### 3.3. Wetting Behavior

**Figure 4:**
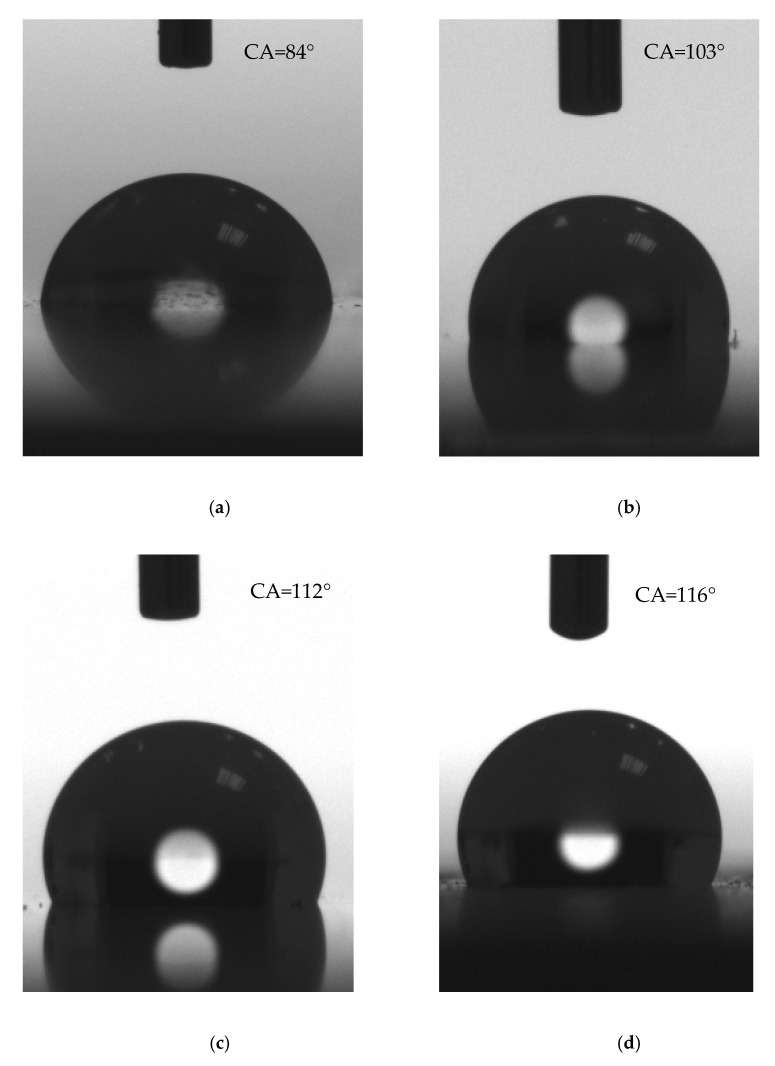
Images of wettability contact angle of the samples. (**a**) reference; (**b**) SiO_2_; (**c**) SiO_2_ + DC88; (**d**) SiO_2_ + POSS14.

The wettability parameters of the samples are presented in Table 3, and some exemplary images of the droplets with the measured contact angles are shown in Figure 4.

The contact angle of the reference sample is a bit lower than 90°. All of the implemented chemical modifications increased the contact angle, resulting in obtaining hydrophobic surfaces. The best water repellent characteristics are observed for Samples 2 and 6, with contact angles equal to 112°±1° and 116°±1°, respectively. For these samples, hybrid modification was introduced (both ${\mathrm{SiO}}_{2}$ and either DC88 or POSS14). Out of the samples with a singular modifier added, the one with POSS14 exhibited the highest value of contact angle.

All of the chemically modified samples are characterized by lower contact angle hysteresis values than the reference sample, which means they are more hydrophobic. Once again, the hybrid modifications resulted in the best outcomes. Furthermore, the values of the sliding angle are also the lowest for Samples 2 and 6, 35°±1° and 30°±1°, respectively, even though satisfying results were also achieved for Samples 4 and 5, around 40°. The highest sliding angle was measured for Sample 3; however, it is also much lower than that of the reference sample.

### 3.4. Ice Accretion

In Table 4 are the collected results of the ice accretion test of the samples tested in three different temperatures, −5 °C,−10 °C and −15 °C. The values of accreted ice and ice reduction are given for each sample.

In the highest temperature, −5 °C, the best results were obtained for the hybrid modification, SiO_2_ + DC88 (Sample 2), as the ice reduction was equal to 65%. However, it should be noted that all of the chemical modifications led to a significant reduction in ice accretion, over 55%.

In the middle temperature, −10 °C, Sample 6 exhibited the highest ice reduction, just below 50%. The worst results were observed for Sample 3, with only an 8% decrease in ice accretion. The samples with POSS14, DC88 and SiO_2_ + DC88 brought in effects similar to each other, around 30% ice reduction.

In the lowest temperature, −15 °C, ice reduction was higher than in −10 °C for Samples 4, 5 and 6. The best results were once again achieved when both SiO_2_ and POSS14 were added (Sample 6) as there was a drop of over 40%. The other modifications resulted in a decrease of approximately 30%, with the worst result for Sample 3.

Comparing ice reduction in different temperatures, chemically modified coatings exhibit the best anti-icing properties when glaze ice is accreted. It is the most challenging to design a coating that would prevent the formation of mixed ice as three out of five samples showed the lowest ice reduction in −10 °C.

## 4. Discussion

### 4.1. Roughness and Wettability

**Figure 5:**
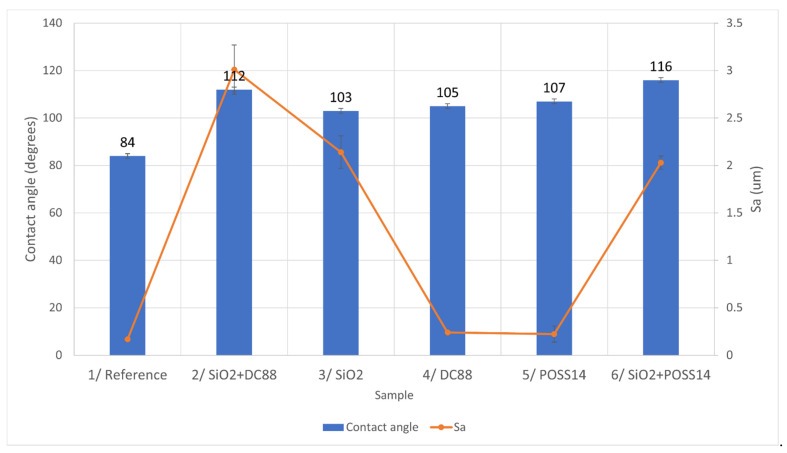
The relationship between roughness of the samples and their wettability.

In Figure 5, the relationship between the roughness and contact angle of the samples is shown.

The samples with hybrid modifications, SiO_2_ + DC88 and SiO_2_ + POSS14, are the most hydrophobic ones. They are quite rough. However, the S_a_ value of Sample 3, modified with only SiO_2,_ is similar to that of Sample 6, yet its contact angle is similar to Samples 4 and 5, which are characterized by smooth surfaces. The possible explanation is that hierarchical structures were achieved for Samples 2 and 6, which resulted in better hydrophobic characteristics.

### 4.2. Relationship between Roughness, Wettability and Ice Reduction

It could be observed in the Results part of this paper that the coatings that achieved the best results in preventing ice accretion of one kind of ice did not necessarily achieve the best outcomes for the other types. Therefore, the rest of this section is divided into three parts in order to discuss in detail the relationship between the tested parameters for glaze, rime and mixed ice.

#### 4.2.1. Glaze Ice

**Figure 6:**
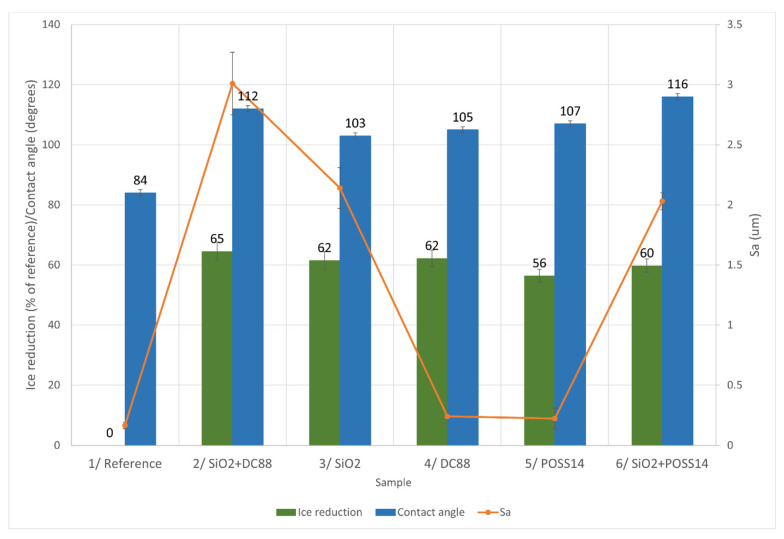
Comparison of different characteristics in aspect of glaze ice reduction.

In Figure 6, the relationship between the roughness of the samples, their wettability and ice reduction in −5 °C is presented.

The highest ice reduction was obtained for Sample 2, which is the roughest one. It also exhibits the second highest contact angle. There seems to be no clear pattern in the aspect of increasing ice reduction by the means of roughness as the values above 62% are achieved by Samples 2, 3 and 4, which are characterized by the highest, medium and lowest values of $S_{a}$. In terms of wettability, Sample 2 exhibits the second highest contact angle. Similar values of contact angle were measured for Samples 3 and 4, which showed equal ice reduction percentages. Having said that, the trend is disrupted by the fact that Sample 6 is the most hydrophobic one, yet its ice reduction is only 60%. However, in the case of glaze ice, the ice reduction values are very close to each other, especially taking into account their standard deviations. Therefore, it is possible that there is actually a direct correlation between wettability and ice accretion that could not be observed due to the measurements’ uncertainty, apart from the fact that the transition from the hydrophilic surface (reference sample) to the hydrophobic one (chemically modified samples) leads to ice reduction of more than 56%. Based on the forming mechanism of glaze ice, it is suggested that the greater portion of the water droplets fell off the coating before freezing, due to the water-repellent behavior of the surface, leading to lower ice accretion.

#### 4.2.2. Mixed Ice

**Figure 7:**
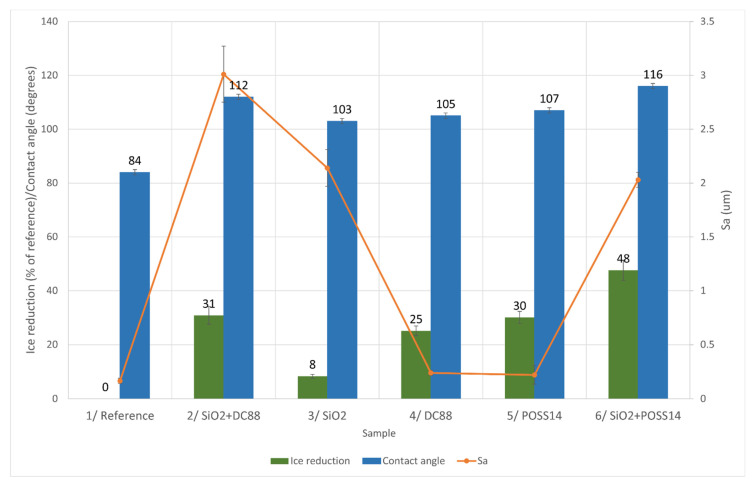
Comparison of different characteristics in aspect of mixed ice reduction.

The influence of roughness and wettability on mixed ice accretion is presented in Figure 7.

Sample 6 is represented by the highest ice reduction, medium roughness and highest contact angle. Once again, the influence of roughness on ice accretion is not clear. Nonetheless, there is a clear correlation between the contact angle and ice reduction values. The conclusion may be drawn that the more hydrophobic the coating is, the more icephobic behavior it exhibits. The dependence is not linear as there is a significant decrease in ice reduction for Sample 3 compared to Sample 4, yet the difference in their contact angle is quite small. Having that said, there is clearly a trend.

#### 4.2.3. Rime Ice

**Figure 8:**
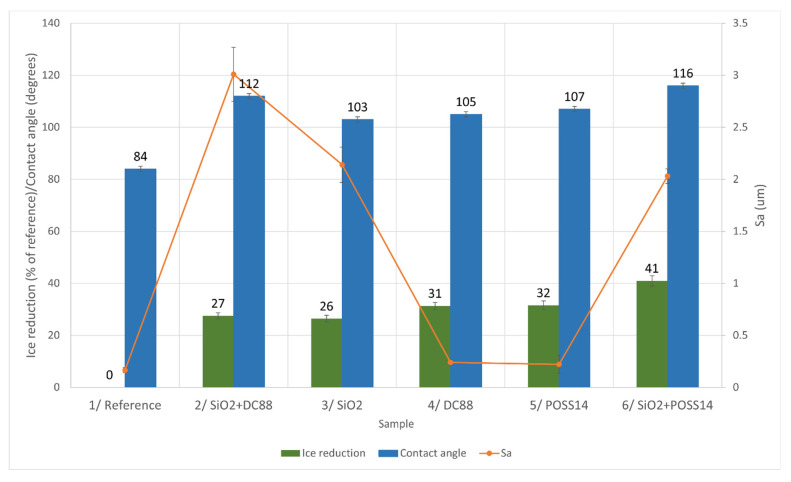
Comparison of different characteristics in aspect of rime ice reduction.

The anti-icing behavior of coatings in −15 °C with rime ice forming was also investigated, as shown in Figure 8.

In the case of rime ice accretion, the most hydrophobic Sample 6 once again exhibits the best anti-icing properties. Moreover, the least hydrophobic Sample 3 is the one with the lowest ice reduction. The wettability of Samples 4 and 5 is similar, and so is their ice reduction value. Both characteristics of Samples 4 and 5 are lower than those of Sample 3. However, the relationship between contact angle and anti-icing cannot be stated as in the case of mixed ice due to the fact that Sample 2 is the second most hydrophobic one yet the second least icephobic. There is no clear correlation between ice accretion and the roughness of the samples.

The fact that there was no correlation found between the roughness of the surfaces and ice accretion in any temperature may lead to a conclusion, presented in Part 4.1. of this paper, that some of the samples (namely 2 and 6) may have hierarchical structures formed, while the others do not have such structures.

## 5. Conclusions

Ice reduction of up to 65% was achieved by the means of the chemical modification of polyurethane coatings. Coatings with the hybrid modifications SiO_2_ + DC88 and SiO_2_ + POSS14 were proven to perform better in terms of ice reduction than coatings with only one modifier. In higher temperatures (−5 °C), i.e., when glaze ice was formed, the coatings with only DC88 or SiO_2_ accreted slightly less ice than the coatings modified with POSS14. However, in lower temperatures, when mixed or rime ice were formed, the sole addition of DC88 or POSS14 led to obtaining much better results than the addition of SiO_2_. Moreover, in temperatures of −10 °C and −15 °C, the best coatings were the ones modified with both SiO_2_ and POSS14, which was manufactured according to the authors’ receipt and is not a commercially available compound. It is also the most hydrophobic coating out of the tested ones. The addition of SiO_2_ causes an increase in surface roughness. Icephobicity is a superposition of the structural effects (caused by SiO_2_) and the effects associated with the introduction of perfluorinated functional groups. The POSS14 additive used in the publication differs from the commercial DOWSIL 88 due to the fact that, in one molecule, we have both functional groups that bind chemically to the SiO_2_ surface (methoxide groups) as well as surface energy modifiers in the form of alkyl and perfluorinated groups. The variation of wettability in terms of the roughness of the samples may lead to the conclusion that a hierarchical structure was formed in the case of the hybrid modifications.

## Figures and Tables

**Table 1:** List of manufactured polyurethane (PUR) nanocomposite coatings.

Sample	SiO_2_ Content (wt %)	DC88 Content (wt %)	POSS14 Content (wt %)
ref	-	-	-
SiO2	3	-	-
DC88	-	5	-
SiO_2_ + DC88	3	5	-
POSS14	-	-	5
SiO_2_ + POSS14	3	-	5

**Table 2:** Roughness parameters of the samples.

Sample Number	Chemical Modification	$S_{a}\;(\mu m)$	$S_{z}\;(\mu m)$
1	Reference	0.166±0.025	5.25±2.64
2	SiO2 + DC88		38.0±9.6
3	SiO2	2.14±0.17	29.7±5.1
4	DC88	0.240±0.024	11.4±2.4
5	POSS14	0.222±0.086	6.37±1.12
6	SiO2 + POSS14	2.03±0.07	20.9±6.2

**Table 3:** Wettability parameters of the samples.

Sample Number	Chemical Modification	Contact Angle (°)	Advancing Contact Angle (°)	Receding Contact Angle (°)	Contact Angle Hysteresis (°)	Sliding Angle (°)
1	Reference	84±1	86±1	47±1	39±2	87±1
2	SiO_2_ + DC88	112±1	117±1	92±1	25±2	35±1
3	SiO_2_	103±1	106±1	75±1	31±2	65±1
4	DC88	105±1	108±1	79±1	29±2	39±1
5	POSS14	107±1	110±1	81±1	29±2	40±1
6	SiO_2_ + POSS14	116±1	120±1	98±1	22±2	30±1

**Table 4:** Results of ice accretion test.

**Sample Number**	Chemical Modification		Accreted Ice (g)		Ice Reduction (% of Reference)		
		−5 °CGlaze Ice	−10 °CMixed Ice	−15 °CRime Ice	−5 °CGLAZE Ice	−10 °CMixed Ice	−15 °CRime Ice
1	Reference	11.34±1.23	9.03±1.62	9.32±1.11	0	0	0
2	SiO2 + DC88	4.36±0.40	8.28±1.60	6.86±0.68	65±3	31±3	27±1
3	SiO2	4.02±0.38	6.25±1.67	6.76±0.24	62±3	8±1	26±1
4	DC88	4.28±0.29	6.77±0.83	6.40±0.42	62±3	25±2	31±1
5	POSS14	4.94±0.15	6.32±0.84	6.38±0.69	56±2	30±2	32±2
6	SiO2 + POSS14	4.56±0.11	4.73±0.71	5.50±0.44	60±2	48±4	41±2
